# Substance use, childhood traumatic experience, and Posttraumatic Stress Disorder in an urban civilian population

**DOI:** 10.1002/da.20751

**Published:** 2010-12

**Authors:** Lamya Khoury, Yilang L Tang, Bekh Bradley, Joe F Cubells, Kerry J Ressler

**Affiliations:** 1 Department of Psychiatry and Behavioral Sciences, Emory University School of Medicine, Atlanta, Georgia; 2 Department of Human Genetics, Emory University School of Medicine, Atlanta, Georgia; 3 Atlanta VA Medical Center, Decatur, Georgia; 4 Howard Hughes Medical Institute, Chevy Chase, Maryland; 5 Yerkes National Primate Research Center, Atlanta, Georgia

**Keywords:** African-American, minority, trauma, childhood maltreatment, psychiatry, alcohol, cocaine, opiate, Marijuana

## Abstract

*Objective*: Exposure to traumatic experiences, especially those occurring in childhood, has been linked to substance use disorders (SUDs), including abuse and dependence. SUDs are also highly comorbid with Posttraumatic Stress Disorder (PTSD) and other mood-related psychopathology. Most studies examining the relationship between PTSD and SUDs have examined veteran populations or patients in substance treatment programs. The present study further examines this relationship between childhood trauma, substance use, and PTSD in a sample of urban primary care patients. *Method*: There were 587 participants included in this study, all recruited from medical and OB/GYN clinic waiting rooms at Grady Memorial Hospital in Atlanta, GA. Data were collected through both screening interviews as well as follow-up interviews. *Results*: In this highly traumatized population, high rates of lifetime dependence on various substances were found (39% alcohol, 34.1% cocaine, 6.2% heroin/opiates, and 44.8% marijuana). The level of substance use, particularly cocaine, strongly correlated with levels of childhood physical, sexual, and emotional abuse as well as current PTSD symptoms. In particular, there was a significant additive effect of number of types of childhood trauma experienced with history of cocaine dependence in predicting current PTSD symptoms, and this effect was independent of exposure to adult trauma. *Conclusions*: These data show strong links between childhood traumatization and SUDs, and their joint associations with PTSD outcome. They suggest that enhanced awareness of PTSD and substance abuse comorbidity in high-risk, impoverished populations is critical to understanding the mechanisms of substance addiction as well as in improving prevention and treatment. Depression and Anxiety, 2010. © 2010 Wiley-Liss, Inc.

## INTRODUCTION

Traumatic life experience, such as physical and sexual abuse as well as neglect, occurs at alarmingly high rates and is considered a major public health problem in the United States.^1^,^2^ Early trauma exposure is well known to significantly increase the risk for a number of psychiatric disorders in adulthood, although many who had childhood trauma exposure are quite resilient. The current study is focused on history of childhood traumatic experiences. Ample evidence has shown that childhood trauma compromises neural structure and function, rendering an individual susceptible to later cognitive deficits and psychiatric illnesses, including schizophrenia, major depression, bipolar disorder, Posttraumatic Stress Disorder (PTSD), and substance abuse.^3^–^8^ Particularly, the link between trauma exposure and substance abuse has been well-established. For example, in the National Survey of Adolescents, teens who had experienced physical or sexual abuse/assault were three times more likely to report past or current substance abuse than those without a history of trauma.^9^ In surveys of adolescents receiving treatment for substance abuse, more than 70% of patients had a history of trauma exposure.^10^,^11^

Furthermore, some studies showed that there is a “dose” or “building block” effect of stress load or trauma on the severity of psychopathology, which is not restricted to PTSD.^12^–^14^ This collection of studies suggest that a simple dose–response model may not be sufficient on its own to explain PTSD risk, but that PTSD diagnosis is likely once an individual passes a certain stress load threshold regardless of other factors. Weber et al.^12^ found that stress load in childhood in particular was related to both the number and severity of depressive and PTSD symptoms in patients with these disorders. Thus, trauma load during the stress-sensitive period of childhood may be especially important when considering psychiatric outcomes. The effects of different types of trauma on psychopathology have also been examined,^15^,^16^ suggesting the effect of trauma may sometimes be type-specific. For example, Powers et al.^15^ found that childhood emotional abuse and neglect were more predictive of adult depression than physical or sexual abuse. Gender may also play an important role in behavioral and psychiatric outcomes of different types of childhood trauma. However, the potential differential role of type of childhood maltreatment on substance abuse in a high-risk population remains unclear.

### COMORBIDITY OF PTSD AND SUBSTANCE USE DISORDER

Studies have also shown that there is high comorbidity between PTSD with substance abuse disorders^3^,^11^,^17^–^20^ and other mental disorders. Breslau et al., in particular, found that exposure to traumatic experience did not increase the risk of substance problems independently of PTSD symptomology. Additionally, evidence has shown that the correlation between trauma and substance abuse is particularly strong for adolescents with PTSD. Up to 59% of young people with PTSD subsequently develop substance abuse problems.^11^,^21^–^23^ This seems to be an especially strong relationship in girls.^24^ Others found that alcohol and drug consumption was associated with greater PTSD symptoms 1 year after a disaster,^25^ Additionally, women who used drugs were found to have significantly higher mean scores for total PTSD symptom severity and were more likely to meet the criteria for a diagnosis of PTSD compared to nonusers.^26^

Early traumatic experience may increase risk of substance use disorders (SUDs) because of attempts to self-medicate or to dampen mood symptoms associated with a dysregulated biological stress response. On the other hand, early adolescent onset of substance use or abuse may further disrupt the biological stress response by increasing plasma cortisol levels, thus additionally contributing to risk for PTSD and comorbid depressive symptoms.^27^ Timing and relative ages of onset are also important when further characterizing this comoribidity between substance abuse and PTSD. Researchers have reported that in cocaine-dependent patients whose PTSD precedes substance abuse, the trauma is most commonly childhood abuse, whereas in those whose substance abuse precedes PTSD onset, the trauma is most commonly associated with the procurement and use of substances.^28^ Some suggest that the comorbidity of PTSD with substance abuse may represent a shared genetically mediated vulnerability to psychopathology after trauma exposure.^24^,^29^

Gender differences in trauma-related risk factors for alcohol and drug abuse have also been reported. One study,^30^ based on data from adolescent samples, suggests that traumatic event exposure increases risk for SUDs for young women, but not young men. Another study^31^ also suggests the existence of a gender difference in comorbidity: in men, drug use preceded the exposure to an event, while in women, the onset age for both drug use and exposure to an event were nearly identical.

The current body of literature regarding substance abuse and PTSD has mostly focused on either military or veteran populations or on treatment-seeking substance-dependent individuals. The current study seeks to extend these findings to a civilian medical population, which will include more females, and to patients who are not associated with a treatment-seeking population for substance use. Additionally, trauma exposure assessments in most of the published studies are relatively simple; questionnaires used in the current study—such as the Early Trauma Inventory (ETI) and the Traumatic Events Inventory (TEI)—can provide more extensive information on trauma history. Finally, most studies report substance use, abuse, or dependence as categorical variables, and few have dealt with the severity of SUDs or with the degree of substance exposure. The current study deals with continuous variables of substance exposure that take into account frequency, duration, and amount used during the period of heaviest use.

In the current study, we examined and extended findings showing the links between childhood trauma exposure, substance use, and PTSD. We assessed indications of a dosage effect of trauma, where higher levels of childhood traumatization might lead to both increased substance use and PTSD symptomology. We hypothesize that, like the findings of Breslau et al.^19^ childhood trauma will not predict substance use independently of PTSD symptoms. However, we do hypothesize that childhood trauma will contribute to increased substance use and PTSD symptoms independently of adult trauma exposure. Finally, we examined evidence of an additive relationship between childhood trauma and substance use problems in predicting the level of PTSD symptomology.

## METHODS

### SUBJECTS/RESEARCH SETTING

All enrolled participants gave written informed consent, and the study was approved by Emory University Institutional Review Board. All potential participants were approached by the research staff at the waiting rooms of the Grady Memorial Hospital General Medical and OB/GYN Clinics, in Atlanta, GA. Subjects in this study were from an ongoing molecular genetics project.^28^,^32–34^ The inclusion criteria were: (1) At least 18 years old, male or female; (2) Able to give informed consent and willing to participate in day of interviews. Exclusion criteria included: (1) Mental retardation (diagnosis in clinic chart); (2) Chart diagnosis of a psychotic disorder. Subjects were reimbursed for their time and effort in the study.

### ASSESSMENTS

All patients who met eligibility criteria and provided consent completed a battery of clinician-administered self-report assessments, which included a demographic form and other basic data, such as subject age, self-identified race, marital status, education, income, and employment. Basic data included, but were not limited to, information related to comorbid psychiatric diagnostic status, family history for psychiatric disorders, past and current substance abuse, stress, and legal issues, etc. To address variation in literacy of participants, all questions were read aloud and answers were recorded by the interviewer. Subjects additionally completed the following interviews:

1 The modified PTSD Symptom Scale (mPSS) is a 17-item interview used to aid in the detection and diagnosis of PTSD symptoms in the 2-week period prior to interview.^28^,^35^,^36^ The structure and content of the mPSS mirror the DSM-IV criteria for PTSD. The psychometric properties of the mPSS indicate that the mPSS has satisfactory internal consistency, high test–retest reliability, and good concurrent validity. The current study examined mPSS total score as well as totals for each symptom cluster.
2 The TEI^37^ is a 14-item screening instrument for lifetime history of traumatic events. For each traumatic event, the TEI assesses experiencing and witnessing separately. It also assesses the confrontation of traumatic events where appropriate. In addition, the TEI also asks the number of times that each event has occurred; age at self-perceived “worst” instance for a given traumatic event; and feelings of helplessness or horror for each traumatic event. The TEI was used in this investigation to assess and control for level of adult trauma exposure.
3 The ETI^38^ evaluates history of childhood physical, sexual, and emotional abuse, and it was administered during follow-up diagnostic interviewing. For each item, age of first occurrence, frequency of occurrence, as well as most common perpetrator is asked. For each type of abuse (physical, sexual, or emotional), scores for total number of types (items endorsed) and total frequency were generated, and these were multiplied to give a comprehensive continuous score for each. The number of types for each of the three abuse types was summed to give a total childhood abuse type score; four quartile groups for childhood trauma were identified based on this total type score.
4 The Kreek–McHugh–Schluger–Kellogg scale (KMSK scale)^39^ quantifies self-exposure to opiates, cocaine, alcohol, tobacco, and/or marijuana use. Each section of the KMSK scale assesses the frequency, amount, and duration of use of a particular substance during the individual's period of greatest consumption (lifetime) and in the 30 days prior to testing (current), and these three values were summed to give lifetime and current total scores. Using a similar sample from the same larger study, total lifetime KMSK scores were tested against dependence diagnoses determined by the Structured Clinical Interview for DSM-IV (SCID) to establish cutoff scores for each substance.^40^ A receiver operating characteristics (ROC) analysis was performed to find the best cutoff score for alcohol, cocaine, opiates, and marijuana dependence. The levels of sensitivity and specificity for each possible cutoff score were determined from the ROC graph, and the cutoff scores with the highest sum total of sensitivity and specificity were found to be the best. Additionally, a χ^2^ analysis was used to find the best cutoff scores; presence or absence of dependence was assigned according to each possible KMSK score for each substance, and these assignments were compared to those determined by SCID interview in a two-by-two contingency table. For alcohol, cocaine, and marijuana, the cutoff scores determined to be best by both ROC analysis and χ^2^ analysis were the same (11, 9, and 8, respectively). The best cutoff score for opiate dependence differed depending on the method (four using sensitivity/specificity analysis and seven using χ^2^ analysis). The more conservative opiate dependence cutoff score of 7 was shown to have a substantially higher positive predictive potential than 4, with only a slight decrease in negative predictive potential (NPP). Thus, the cutoff scores determined by these methods for alcohol (11), cocaine (9), marijuana (8), and heroin/opiates (7) determined the dependence groups used in the current study.
5 Beck Depressive Inventory (BDI) is a 21-item interview used to detect the presence of depressive symptoms in the 2-week period prior to testing.^41^ Each item is rated on the severity of that specific symptom. The current study uses the BDI total score variable in certain analyses to control for the presence of current depressive symptoms.

### ANALYSIS

All analyses were performed using SPSS 17.0 software. Descriptive statistics on demographics were calculated and expressed in terms of the total number of subjects and percentages of the sample as a function of gender and a particular characteristic. Gender differences for demographic variables and measure characteristics were determined using student *t*-tests and χ^2^ analyses where appropriate. We used two-tailed Pearson's correlations to show the associations between severity of childhood trauma exposure and levels of substance exposure and PTSD symptoms. Univariate analyses were used to examine differences in PTSD symptom level between substance dependence groups, as well as between the childhood trauma quartile groups. Further univariate analyses examined trends in substance exposure across the four childhood trauma groups, with post-hoc analyses controlling for adult trauma exposure and PTSD symptomology.

## RESULTS

### SAMPLE CHARACTERISTICS

A total of 587 participants were included in this study, with a greater number of females (*N* = 359, 61.2%) than males (*N* = 228, 38.8%). Table 1 shows demographic information for the entire sample as well as the significant differences between males and females. The mean and standard deviations of the main outcome variables in this sample are also indicated (Table 2).

**Table 1:** Demographics

	Total sample *N* = 587	Males *N* = 228	Females *N* = 359
Age***—mean (*SD*)	42.35 (12.72)	45.42 (10.96)	40.39 (13.38)
Race/ethnicity	*N* = 578	*N* = 224	*N* = 354
Black	527 (91.2)	203 (90.6)	324 (91.5)
Non-black	51 (8.8)	21 (9.4)	30 (8.5)
Education*	*N* = 577	*N* = 224	*N* = 353
<12th Grade	136 (23.8)	37 (16.5)	99 (28.0)
High school/GED	240 (41.5)	100 (44.6)	140 (39.7)
Some college/tech	130 (22.5)	54 (24.1)	76 (21.5)
Tech school grad	23 (4.0)	9 (4.0)	14 (4.0)
College grad or higher	48 (8.4)	24 (10.7)	24 (6.8)
Relationship status***	*N* = 575	*N* = 223	*N* = 352
Single, never married	331 (57.6)	118 (52.9)	213 (60.5)
Married/domestic partner	67 (11.6)	33 (14.8)	34 (9.7)
Divorced	100 (17.4)	54 (24.2)	46 (13.1)
Separated	45 (7.8)	17 (7.6)	28 (8.0)
Widowed	32 (5.6)	1 (0.4)	31 (8.8)
Monthly income	*N* = 564	*N* = 221	*N* = 343
<1,000	405 (71.8)	162 (73.3)	244 (71.1)
1,000–1,999	111 (19.7)	39 (17.6)	72 (21.0)
>2,000	48 (8.5)	21 (9.5)	27 (7.9)
Currently unemployed*	449/578 (77.7)	185/224 (82.6)	264/354 (74.6)
Current Disability Support*	149/576 (25.9)	70/222 (31.5)	79/354 (22.3)
Ever been Arrested***	370/577 (64.1)	187/224 (83.5)	183/353 (51.8)
Ever been in jail***	347/577 (60.1)	175/224 (78.1)	172/353 (48.7)
Ever been in prison***	96/574 (16.7)	70/223 (31.4)	26/351 (7.4)
Ever had psychiatric hospitalization	108/573 (18.8)	40/222 (18.0)	68/351 (19.4)

N/Total *N* for each item (%) for each demographic variable.

* *P*<.05;

*** *P*<.001 for gender differences.

**Table 2:** Measure characteristics: mean (*SD*) for each variable

	Total sample	Males	Females
KMSK total scores			
Alcohol (lifetime)***	7.99 (4.47)	9.72 (3.61)	6.84 (4.62)
Alcohol (current)*	2.70 (3.68)	3.35 (4.45)	2.33 (3.27)
Cocaine (lifetime)***	5.22 (6.50)	6.87 (6.62)	4.18 (6.22)
Cocaine (current)	0.15 (1.03)	0.29 (1.54)	0.06 (0.54)
Heroin/opiate (lifetime)**	0.81 (2.48)	1.24 (3.08)	0.54 (1.96)
Heroin/opiate (current)*	0.13 (1.25)	0.34 (2.05)	0 (0)
Marijuana (lifetime)***	6.01 (5.13)	7.70 (5.03)	5.14 (4.96)
Marijuana (current)	1.23 (2.92)	1.28 (3.13)	1.19 (2.80)
Tobacco (lifetime)**	6.93 (4.90)	7.72 (4.63)	6.42 (5.0)
Tobacco (current)	3.10 (3.80)	3.67 (3.83)	2.77 (3.76)
ETI type*frequency scores			
Physical Abuse	35.41 (49.49)	39.45 (54.49)	32.85 (45.93)
Sexual abuse**	33.15 (84.99)	21.47 (64.79)	40.58 (94.97)
Emotional abuse*	55.21 (74.50)	47.40 (69.46)	60.17 (77.22)
MPSS scores			
Total	13.25 (12.32)	12.48 (12.13)	13.76 (12.44)
Intrusive	3.19 (3.83)	2.81 (3.66)	3.43 (3.92)
Avoidance/numbing	5.40 (5.52)	5.34 (5.51)	5.43 (5.54)
Hyperarousal	4.70 (4.46)	4.36 (4.25)	4.92 (4.59)

* *P*<.05;

** *P*<.01

*** *P*<.001 for gender differences.

Rates of lifetime substance dependence, as determined by KMSK cutoff scores, were high in this sample. Marijuana was the most common substance of abuse with 44.8% of a subset of 373 participants who completed that section of the questionnaire falling in the dependency group. Alcohol was the next most common (39%), followed by cocaine (34.1%), and then heroin/opiates (6.2%).

### CHILDHOOD TRAUMA AND SUBSTANCE USE

Table 3 demonstrates a strong association between adverse childhood experience (type×frequency score) and levels of exposure to various substances both currently and during the period of heaviest use. Gender differences in substance use correlates of the different types of childhood abuse are also observed. In women, sexual abuse was significantly linked to lifetime cocaine (*P*<.001) as well as marijuana exposure (*P*<.01). Physical abuse in men significantly correlates with current cocaine and lifetime/current heroin use (*P*<.01), while in women it is linked to lifetime cocaine and marijuana use (*P*<.01). Emotional abuse in men significantly correlates to current heroin exposure (*P*<.01), whereas in women it is linked to heavier lifetime cocaine use (*P*<.01).

**Table 3:** Correlations between childhood abuse type*frequency score and substance use

	All	Males	Females
Physical abuse			
Alcohol (life) ♯	*r* = .110**	*r* = .065	*r* = .112
Alcohol (current	*r* = .010		
Cocaine (life) ♯	*r* = .156***	*r* = .136	*r* = .155**
Cocaine (current)	*r* = .190**		
Heroin/Opiate (life)♯	*r* = .123**	*r* = .207**	*r* = .003
Heroin/Opiate (current) ♯	*r* = .251***	*r* = .352**	
Marijuana (life)♯	*r* = .199***	*r* = .186	*r* = .196**
Marijuana (current) ♯	*r* = .043	*r* = .154	*r* = −.048
Tobacco (life) ♯	*r* = .148***	*r* = .053	*r* = .205***
Tobacco (current)	*r* = .031		
Sexual abuse			
Alcohol (life) ♯	*r* = .081	*r* = .103	*r* = .128
Alcohol (current)	*r* = −.129		
Cocaine (life) ♯	*r* = .127**	*r* = −.016	*r* = .235***
Cocaine (current)	*r* = .004		
Heroin/Opiate (life) ♯	*r* = .038	*r* = .019	*r* = .085
Heroin/Opiate (current) ♯	*r* = −.011	*r* = .014	
Marijuana (life) ♯	*r* = .146**	*r* = .071	*r* = .216**
Marijuana (current) ♯	*r* = −.039	*r* = −.098	*r* = −.008
Tobacco (life) ♯	*r* = .129**	*r* = .054	*r* = .184***
Tobacco (current)	*r* = −.018		
Emotional abuse			
Alcohol (life) ♯	*r* = .093	*r* = .098	*r* = .140
Alcohol (current)	*r* = −.069		
Cocaine (life) ♯	*r* = .108**	*r* = .109	*r* = .140**
Cocaine (current)	*r* = .071		
Heroin/Opiate (life) ♯	*r* = .045	*r* = .153	*r* = −.029
Heroin/Opiate (current) ♯	*r* = .144	*r* = .285**	
Marijuana (life) ♯	*r* = .115	*r* = .142	*r* = .142
Marijuana (current)	*r* = −.005		
Tobacco (life) ♯	*r* = .137**	*r* = .053	*r* = .201***
Tobacco (current)	*r* = .021		

** *P*<.01;

*** *P*<.001;

♯ *P*<.05 for child abuse×gender interaction term. Only those with significant interaction were stratified by gender.

Analysis of childhood trauma quartiles, which combined all three types of abuse, demonstrated increased levels of lifetime alcohol (*F* = 5.97, *P*<.001), cocaine (*F* = 3.90, *P*<.01)*,* and marijuana (*F* = 9.18, *P*<.001) exposure with increased trauma load (Fig. .1). Significant group differences between specific quartiles are indicated. These analyses controlled for age and sex; when adult trauma exposure was introduced as a covariate, only the increases in alcohol (*F* = 2.92, *P*<.05) and marijuana use (*F* = 5.162, *P*<.01) remained statistically significant. The increase in these two substances additionally remained significant after independently controlling for current PTSD symptom level (Alcohol: *F* = 3.61, *P*<.05; Marijuana: *F* = 6.57, *P*<.001). While heroin exposure did appear to increase overall across the four quartiles as well, this trend did not reach statistical significance. However, a significant group difference in heroin exposure was observed between the second and fourth quartiles.

**1:**
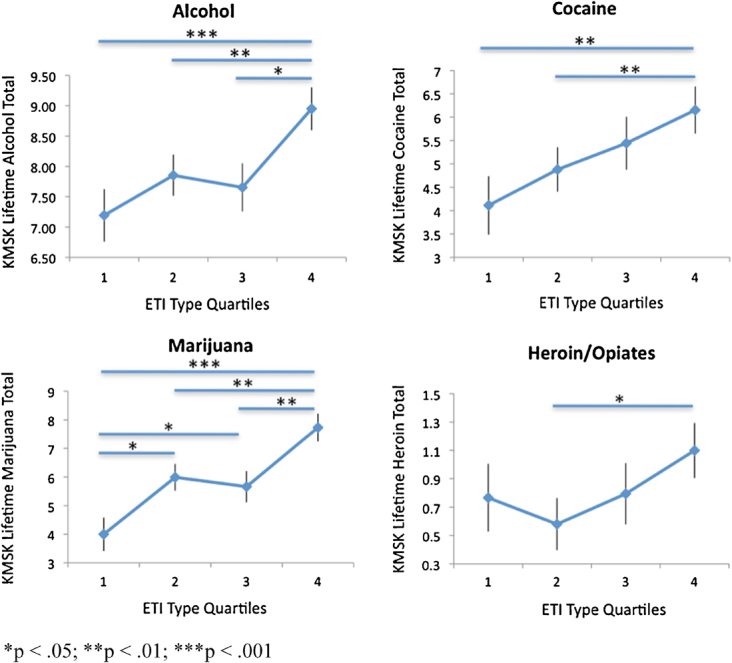
Substance use across childhood trauma quartiles. Of these four substances, alcohol (*F* = 5.97, *P*<.001), cocaine (*F* = 3.90, *P*<.01) and marijuana use (*F* = 9.18, *P*<.001) increased significantly across the four childhood trauma quartiles. Significant group differences in KMSK scores between specific quartiles are indicated on each graph. These data suggest a dosage effect of childhood trauma load on substance exposure, particularly alcohol, cocaine, and marijuana, later on. KMSK, Kreek–McHugh–Schluger–Kellogg.

### PTSD AND SUBSTANCE DEPENDENCE

Differential levels of current PTSD symptomology between those with and without lifetime substance dependence are demonstrated in Figure 2. After controlling for age and sex, lifetime cocaine dependence was significantly associated with a higher PSS total score (*F* = 26.90, *P*<.001) as well as symptom level across all three clusters (Intrusive: *F* = 18.46, *P*<.001; Avoidance/Numbing: *F* = 20.91, *P*<.001; and Hyperarousal: *F* = 23.07, *P*<.001). Lifetime marijuana dependence was also associated with PSS total (*F* = 10.12, *P*<.01) and symptoms across all clusters (Intrusive: *F* = 4.16, *P*<.05; Avoidance/Numbing: *F* = 11.25, *P*<.01; and Hyperarousal: *F* = 7.72, *P*<.01). Lifetime alcohol dependence was associated with PSS total (*F* = 6.48, *P*<.05), avoidance/numbing (*F* = 6.92, *P*<.01), and hyperarousal symptoms (*F* = 4.46, *P*<.05). Lifetime heroin dependence was not significant in predicting current PTSD levels. After controlling for current level of depressive symptoms, only the marijuana dependence group differences between PSS total, intrusive, and hyperarousal scores remained significant. No other substance dependence group differences were significant after depressive symptoms were taken into account.

**2:**
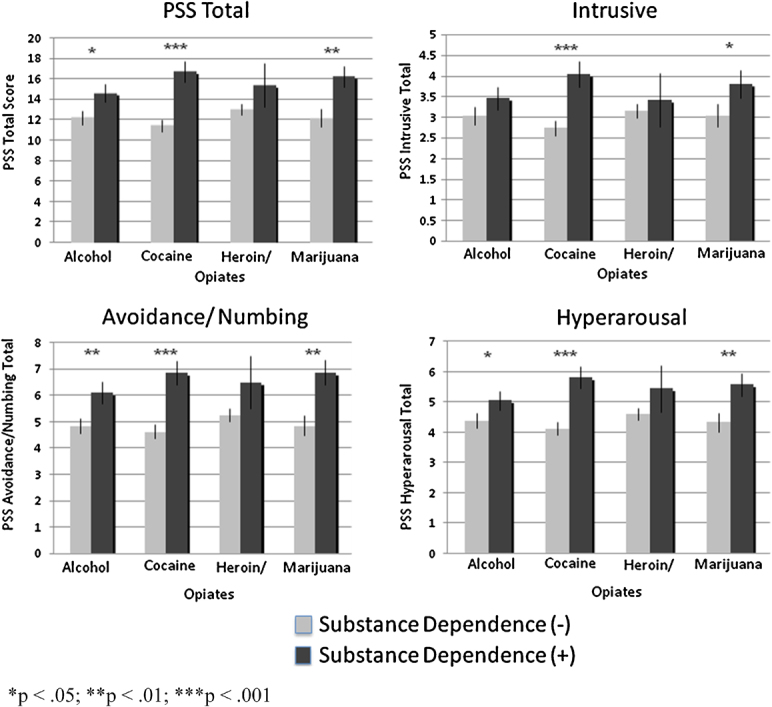
PTSD symptoms between substance dependence groups. These graphs show differences between PTSD symptom level between alcohol, cocaine, and marijuana-dependent and non-dependent groups. While the differences for cocaine and marijuana dependence applied across all symptom clusters, those for alcohol dependence applied to all but intrusive symptoms. PTSD, Posttraumatic Stress Disorder.

### CHILDHOOD TRAUMA, SUBSTANCE DEPENDENCE, AND PTSD

Using a two-tailed Pearson correlation, the total number of types of childhood trauma correlated significantly with current total PTSD symptoms in this sample (*r* = .399, *P*<.001). Childhood trauma quartile analyses demonstrate increased levels of PTSD symptomology, both in PSS total score (*F* = 27.92, *P*<.001) as well as across the symptom clusters (Intrusive: *F* = 18.43, *P*<.001; Avoidance/Numbing: *F* = 25.18, *P*<.001; Hyperarousal: *F* = 19.56, *P*<.001) with higher level of childhood trauma exposure. These relationships remained significant after controlling for age, sex, and level of adulthood trauma exposure.

Further analyses on the effect of childhood trauma load on current PTSD symptoms took into account substance dependence history. Across all four quartiles, history of cocaine dependence was associated with higher PSS scores (Fig. .3; *F* = 13.50, *P*<.001). This relationship remained significant after controlling for age, sex, and adulthood trauma exposure. However, this relationship was no longer significant after current depressive symptomology was included in the model. Closer examination of each quartile showed significant substance dependence group differences in mean PSS score at the second (*F* = 6.66, *P*<.05), third (*F* = 4.13, *P*<.05), and fourth (*F* = 7.43, *P*<.01) quartiles only.

**3:**
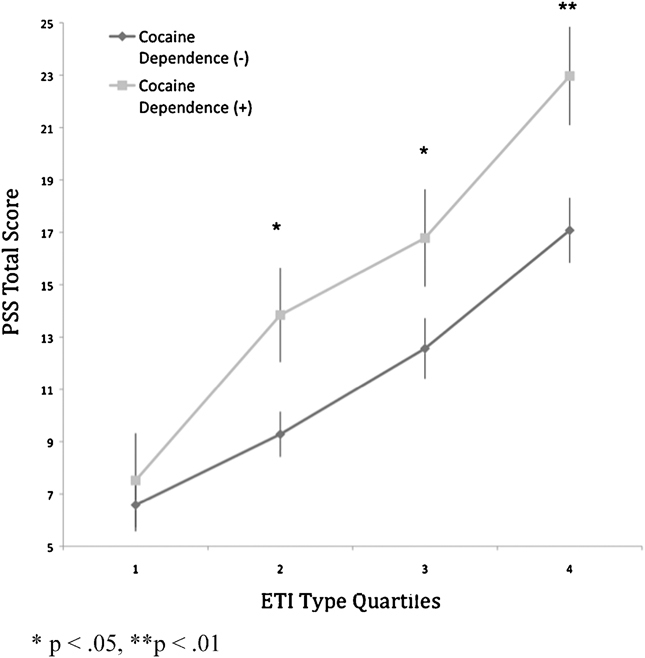
Current PTSD level between cocaine dependence groups across childhood trauma quartiles. This graph indicates an additive effect of childhood trauma load and past cocaine dependence in predicting current PTSD symptom level. Across all four quartiles, the cocaine-dependent group had significantly higher PSS scores than the non-dependent group (*F* = 13.50, *P*<.001). Significant group differences at the second, third and fourths quartiles are also indicated. PTSD, Posttraumatic Stress Disorder; PSS, PTSD Symptom Scale.

## DISCUSSION

The current study confirms previous findings of a strong relationship between adverse childhood experience and subsequent substance use and poor mental health outcomes, particularly PTSD.^42^ In all subjects, physical abuse correlated with the use of all substances examined, while sexual abuse in childhood associated with cocaine and marijuana use only, suggesting differential effects of abuse type on substance use. The findings with regard to sexual abuse appear to be driven by significant associations in women but not men; this is consistent with the higher prominence of childhood sexual abuse in women in this sample. Additionally, emotional abuse was associated with cocaine use in the current study.

Examination of the childhood trauma quartiles shows alcohol, cocaine, and marijuana use significantly increasing across the four quartiles. This essentially indicates a progressive effect of trauma load on the severity of use of these particular substances. While heroin use did not increase significantly across the childhood trauma quartiles overall, group differences were observed between the second and fourth quartiles, indicating a trend in that direction. It is important to consider that these childhood trauma quartiles represent the number of types of childhood abuse experienced; other important factors may include severity and frequency of abuse, age of first occurrence, as well as perpetrator identity.

Although we predicted that this effect of multiple traumatization would not be independent of PTSD symptoms, alcohol and marijuana (but not cocaine) use still increased significantly across childhood trauma quartiles even after controlling for PTSD. Other researchers^43^ have found PTSD to be a significant mediator of the effect of childhood abuse on substance use problems later on, and we similarly found that PTSD symptoms may account for cocaine use in individuals who have experienced childhood trauma. The absence of this finding for other substances could be accounted for by the different time periods assessed; since we looked at lifetime substance exposure but current PTSD symptoms, it is possible that the use of alcohol or marijuana may have been better accounted for by PTSD symptoms occurring at the same time, or several years before the onset of substance use problems as findings by Douglas et al.^44^ suggest. Additionally, as hypothesized, childhood trauma contributed to increased alcohol and marijuana use independently of adult trauma exposure. However, the effect of childhood trauma load on cocaine use was not independent of adult trauma, which may be indicative of adult trauma in this population that is associated with obtaining and using this particular substance.

A progressive effect of childhood trauma load on PTSD symptomology was also observed, where childhood trauma contributes to higher total PTSD symptoms as well as higher levels of symptoms in each cluster. The effect of childhood trauma on PTSD severity was also found to be independent of adult trauma. When substance dependence was taken into account, only cocaine dependence showed a significant additive relationship with childhood trauma in predicting PTSD severity. It was also the cocaine-dependent group that scored significantly higher in PTSD scores across all clusters. However, these findings were no longer significant after controlling for current depressive symptoms, perhaps reflecting the high comorbidity between PTSD and depression as well as a strong relationship between depression and substance use problems. The strong association between cocaine dependence and PTSD symptoms may in part be due to the nature of the drug itself; as a stimulant, cocaine use may contribute to and enhance hyperarousal symptoms in particular. These findings can also be understood in the context of a high prevalence of crack cocaine use in this population. While marijuana use is also extraordinarily prevalent, and marijuana dependence did predict higher PTSD scores across all clusters, caution must be used in interpreting these results; the KMSK cutoff score determined for marijuana dependence was the first to be established for this substance, thus it needs to be validated further before we can know how useful it is.

Several limitations exist with respect to this study. As with all similar studies of adult retrospective reporting of child maltreatment histories, we cannot rule out possibilities of recall bias in these subjective reports. Furthermore, we do not currently have sufficient data with regards to the timing of the trauma, PTSD symptoms, and substance abuse histories, so that these data are correlative, but cannot imply direction of causation. We believe that these effects are generalizable to urban, traumatized civilian populations at high risk for substance abuse, but perhaps not to other populations. Especially given the extremely high percentage of African Americans in this sample (91.2%), these findings may not be generalizable to populations with different racial profiles.

In summary, we find that there are high rates of lifetime dependence on various substances in this high-risk population. Additionally, the level of substance use, particularly cocaine, strongly associated with levels of childhood physical, sexual, and emotional abuse as well as current PTSD symptoms. There was a significant additive effect of number of types of childhood trauma experienced with lifetime cocaine dependence in predicting current PTSD symptoms, and this effect was independent of levels of adult trauma. These data suggest that enhanced awareness of the comorbidity between PTSD and substance abuse is critical both in understanding mechanisms of substance addiction as well as in improving prevention and treatment.

## References

[1] American Psychological Association (2002). Violence and the Family: Report of the American Psychological Association Presidential Task Force on Violence and the Family.

[2] Costello EJ, Erkanli A, Fairbank JA, Angold A (2002). The prevalence of potentially traumatic events in childhood and adolescence. J Trauma Stress.

[3] Mills KL, Teesson M, Ross J, Peters L (2006). Trauma, PTSD, and substance use disorders: findings from the Australian National Survey of Mental Health and Well-Being. Am J Psychiatry.

[4] Dube SR, Anda RF, Felitti VJ, Edwards VJ, Croft JB (2002). Adverse childhood experiences and personal alcohol abuse as an adult. Addict Behav.

[5] Kingston S, Raghavan C (2009). The relationship of sexual abuse, early initiation of substance use, and adolescent trauma to PTSD. J Trauma Stress.

[6] Arnow BA (2004). Relationships between childhood maltreatment, adult health and psychiatric outcomes, and medical utilization. J Clin Psychiatry.

[7] Gillespie CF, Nemeroff CB (2005). Early life stress and depression. Curr Psychiatry.

[8] Schneider R, Baumrind N, Kimerling R (2007). Exposure to child abuse and risk for mental health problems in women. Violence Vict.

[9] Kilpatrick DG, Saunders BE, Smith DW (2003). http://www.ncjrs.gov/pdffiles1/nij/194972.pdf.

[10] Funk RR, McDermeit M, Godley SH, Adams L (2003). Maltreatment issues by level of adolescent substance abuse treatment: the extent of the problem at intake and relationship to early outcomes. Child Maltreat.

[11] Deykin EY, Buka SL (1997). Prevalence and risk factors for posttraumatic stress disorder among chemically dependent adolescents. Am J Psychiatry.

[12] Weber K, Rockstroh B, Borgelt J (2008). Stress load during childhood affects psychopathology in psychiatric patients. BMC Psychiatry.

[13] Neuner F, Schauer M, Karunakara U, Klaschik C, Robert C, Elbert T (2004). Psychological trauma and evidence for enhanced vulnerability for posttraumatic stress disorder through previous trauma among West Nile refugees. BMC Psychiatry.

[14] Kaysen D, Rosen G, Bowman M, Resick PA (2009). Duration of exposure and the dose-response model of PTSD. J Interpers Violence.

[15] Powers A, Ressler KJ, Bradley RG (2009). The protective role of friendship on the effects of childhood abuse and depression. Depress Anxiety.

[16] Garnefski N, Diekstra RF (1997). Child sexual abuse and emotional and behavioral problems in adolescence: gender differences. J Am Acad Child Adolesc Psychiatry.

[17] Reynolds M, Mezey G, Chapman M, Wheeler M, Drummond C, Baldacchino A (2005). Co-morbid post-traumatic stress disorder in a substance misusing clinical population. Drug Alcohol Depend.

[18] Najavits LM, Gastfriend DR, Barber JP (1998). Cocaine dependence with and without PTSD among subjects in the National Institute on Drug Abuse Collaborative Cocaine Treatment Study. Am J Psychiatry.

[19] Breslau N, Davis GC, Schultz LR (2003). Posttraumatic stress disorder and the incidence of nicotine, alcohol, and other drug disorders in persons who have experienced trauma. Arch Gen Psychiatry.

[20] Reed PL, Anthony JC, Breslau N (2007). Incidence of drug problems in young adults exposed to trauma and posttraumatic stress disorder: do early life experiences and predispositions matter?. Arch Gen Psychiatry.

[21] Clark DB, Lesnick L, Hegedus AM (1997). Traumas and other adverse life events in adolescents with alcohol abuse and dependence. J Am Acad Child Adolesc Psychiatry.

[22] Giaconia RM, Reinherz HZ, Hauf AC, Paradis AD, Wasserman MS, Langhammer DM (2000). Comorbidity of substance use and post-traumatic stress disorders in a community sample of adolescents. Am J Orthopsychiatry.

[23] Perkonigg A, Kessler RC, Storz S, Wittchen HU (2000). Traumatic events and post-traumatic stress disorder in the community: prevalence, risk factors and comorbidity. Acta Psychiatr Scand.

[24] Lipschitz DS, Rasmusson AM, Anyan W (2003). Posttraumatic stress disorder and substance use in inner-city adolescent girls. J Nerv Ment Dis.

[25] Kishore V, Theall KP, Robinson W (2008). Resource loss, coping, alcohol use, and posttraumatic stress symptoms among survivors of Hurricane Katrina: a cross-sectional study. Am J Disaster Med.

[26] Sullivan TP, Holt LJ (2008). PTSD symptom clusters are differentially related to substance use among community women exposed to intimate partner violence. J Traumatic Stress.

[27] DeBellis M (2002). Developmental traumatology: a contributory mechanism for alcohol and substance use disorders. Psychoneuroendocrinology.

[28] Brady KT, Dansky BS, Sonne SC, Saladin ME (1998). Posttraumatic stress disorder and cocaine dependence. Order of onset. Am J Addict.

[29] Scherrer JF, Xian H, Lyons MJ (2008). Posttraumatic stress disorder; combat exposure; and nicotine dependence, alcohol dependence, and major depression in male twins. Compr Psychiatry.

[30] Danielson CK, Amstadter AB, Dangelmaier RE, Resnick HS, Saunders BE, Kilpatrick DG (2009). Trauma-related risk factors for substance abuse among male versus female young adults. Addict Behav.

[31] Cottler LB, Nishith P, Compton WM (2001). Gender differences in risk factors for trauma exposure and post-traumatic stress disorder among inner-city drug abusers in and out of treatment. Compr Psychiatry.

[32] Binder EB, Bradley RG, Liu W (2008). Association of FKBP5 polymorphisms and childhood abuse with risk of posttraumatic stress disorder symptoms in adults. J Am Med Assoc.

[33] Bradley RG, Binder EB, Epstein MP (2008). Influence of child abuse on adult depression: moderation by the corticotropin-releasing hormone receptor gene. Arch Gen Psychiatry.

[34] Gillespie CF, Bradley B, Mercer K (2009). Trauma exposure and stress-related disorders in inner city primary care patients. Gen Hosp Psychiatry.

[35] Foa EB, Riggs DS, Dancu CV, Rothbaum BO (1993). Reliability and validity of a brief instrument for assessing posttraumatic stress disorder. J Trauma Stress.

[36] Foa EB, Tolin DF (2000). Comparison of the PTSD symptom scale-interview version and the clinician-administered PTSD scale. J Trauma Stress.

[37] Schwartz AC, Bradley RL, Sexton M, Sherry A, Ressler KJ (2005). Posttraumatic stress disorder among African Americans in an inner city mental health clinic. Psychiatr Serv.

[38] Bremner JD, Vermetten E, Mazure CM (2000). Development and preliminary psychometric properties of an instrument for the measurement of childhood trauma: the Early Trauma Inventory. Depress Anxiety.

[39] Kellogg SH, McHugh PF, Bell K (2003). The Kreek-McHugh-Schluger-Kellogg scale: a new, rapid method for quantifying substance abuse and its possible applications. Drug Alcohol Depend.

[40] Tang Y, Khoury L, Bradley B, Gillespie CF, Ressler KJ, Cubells JF (2010). Substance use disorders assessed using the Kreek-McHugh-Schluger-Kellogg (KMSK) scale in an urban low-income and predominantly African-American sample of primary care patients. Am J Addict.

[41] Beck AT, Ward CH, Mendelson M, Mock J, Erbaugh J (1961). An inventory for measuring depression. Arch Gen Psychiatry.

[42] Wu NS, Schairer LC, Dellor E, Grella C (2010). Childhood trauma and health outcomes in adults with comorbid substance abuse and mental health disorders. Addict Behav.

[43] White HR, Widom CS (2008). Three potential mediators of the effects of child abuse and neglect on adulthood substance use among women. J Stud Alcohol Drugs.

[44] Douglas KR, Chan G, Gelernter J (2010). Adverse childhood events as risk factors for substance dependence: partial mediation by mood and anxiety disorders. Addict Behav.

